# Type 1 fimbriae are important factors limiting the dissemination and colonization of mice by *Salmonella* Enteritidis and contribute to the induction of intestinal inflammation during *Salmonella* invasion

**DOI:** 10.3389/fmicb.2015.00276

**Published:** 2015-04-09

**Authors:** Marta Kuźmińska-Bajor, Krzysztof Grzymajło, Maciej Ugorski

**Affiliations:** ^1^Department of Biotechnology and Food Microbiology, Wrocław University of Environmental and Life Sciences, WrocławPoland; ^2^Department of Biochemistry, Pharmacology and Toxicology, Faculty of Veterinary Medicine, Wrocław University of Environmental and Life Sciences, WrocławPoland; ^3^Laboratory of Glycobiology and Cell Interactions, Ludwik Hirszfeld Institute of Immunology and Experimental Therapy, Polish Academy of Sciences, WrocławPoland

**Keywords:** *Salmonella* Enteritidis, type 1 fimbriae, FimH, adhesion, pathogenicity, innate immune response, mice incection, pro-inflammatory interleukins

## Abstract

We have recently shown that *Salmonella* Gallinarum type 1 fimbriae with endogenous mannose-resistant (MR) variant of the FimH protein increase systemic dissemination of *S.* Gallinarum and colonization of internal organs in comparison to the *S*. Gallinarum *fimH* knockout strain or the mutant expressing mannose-sensitive (MS) FimH variant from *S*. Enteritidis. Elaborating from these studies, we proposed that MS variants of FimH are advantageous in gastrointestinal infections, in contrast to MR FimH variants which decrease intestinal colonization and promote their systemic spreading. To support our hypothesis, we carried out *in vivo* studies using mice infected with wild-type *S.* Enteritidis and its *fimH* knockout strain (*S*. Enteritidis), which was characterized by significantly lower adhesion and invasiveness of murine ICE-1 intestinal cells. Using bioluminescence imaging, we observed that the loss of MS FimH adhesin correlates well with the highly increased colonization of mice by these bacteria. The appearance of the mutant strain was observed much earlier than wild-type *Salmonella*, and mice infected with 10^4^–10^7^ *S*. Enteritidis fimH::kan CFUs had significantly (*P* < 0.05) shorter infection-free time than animals inoculated with wild-type *S.* Enteritidis. Infections caused by non-typhoid *Salmonella,* such as *S*. Enteritidis, are associated with massive inflammation of the lamina propria and lymph nodes in the intestinal tract. Therefore, we evaluated the role of MS type 1 fimbriae in the induction of cytokine expression and secretion, using murine ICE-1 intestinal cells. We showed that the expression, as well as secretion, of *Il-1b*, *Il-6*, *Il-10*, and *Il-12b* was significantly higher in cells infected with wild-type *S*. Enteritidis compared to cells infected with the mutant strain. Based on our results, we propose that type 1 fimbriae may play an important role in the pathogenicity of *S*. Enteritidis and may contribute to an intestinal inflammatory response.

## Introduction

Increasing evidence suggests that type 1 fimbriae play an important role in the initial stages of *Salmonella* infections as this has been shown using different animal models (Lockman and Curtiss, 1992; van der Velden et al., 1998; Dibb-Fuller et al., 1999; Dibb-Fuller and Woodward, 2000; De Buck et al., 2004). However, their exact role in these processes is still controversial (Allen-Vercoe and Woodward, 1999a,b; Rajashekara et al., 2000). Type 1 fimbriae are proteinaceous filamentous structures present on the surface of many members of *Enterobacteriaceae*, including the genus *Salmonella*, which promote bacterial adhesion to host tissues (Duguid et al., 1976). They are composed primarily of protein subunits called FimA. Notwithstanding, the adhesive properties of type 1 fimbriae depend on a lectin-like FimH adhesin located on the top of the fimbrial shaft (Krogfelt et al., 1990). By reason of type 1 fimbriae of most *Salmonella* serovars bind to oligomannosidic structures present on eukaryotic glycoproteins and these interactions are inhibited by D-mannose, they are called mannose-sensitive (MS; Duguid et al., 1976; Firon et al., 1984). MS type 1 fimbriae mediate a bacterial adhesion to, and colonization of gut mucosa in different animal hosts (Lindquist et al., 1987; Dibb-Fuller and Woodward, 2000; Robertson et al., 2000; Althouse et al., 2003). Nonetheless, the specific glycoprotein counter-receptors carrying high-mannose oligosaccharides expressed by enterocytes are presently not known. Bearing that in mind, we have recently shown that FimH adhesin from *S*. Enteritidis binds to enterocyte membrane protein of 130 kDa, and FimH adhesins from *S*. Abortusovis, *S*. Choleraesuis and *S*. Dublin bind to membrane protein of about 55 kDa (Grzymajlo et al., 2013). MS type 1 fimbriae are also involved in bacterial transcytosis through M-cells, where surface glycoprotein GP2 acts as a receptor for FimH adhesion (Hase et al., 2009; Ohno and Hase, 2010; Peterson and Artis, 2014).

All broad-host range *Salmonella* serovars (e.g., *S.* Enteritidis and *S.* Typhimurium), typically producing a self-limiting gastroenteritis, express mannose-ensitive FimH adhesins. In contrast, type 1 fimbrial FimH proteins of the majority of host-restricted *Salmonella* serovars (e.g., *S.* Gallinarum), causing systemic disease with enteric fever and bacteremia (Mizel et al., 1995), have been found to be deprived of MS binding (mannose-resistant, MR), and show no activity toward various eukaryotic cells (Duguid et al., 1976; Hancox et al., 1997; Guo et al., 2009; Kisiela et al., 2012). These divergences are associated with differences in amino acid sequences of FimH adhesins (Hancox et al., 1997; Kisiela et al., 2012). Since such mutations are also found among MS FimH adhesins, they may be partially responsible for the tropism of various *Salmonella* serovars to different species, tissues and cells (Boddicker et al., 2002; Kuzminska-Bajor et al., 2012; Grzymajlo et al., 2013).

We have recently shown that *S*. Gallinarum type 1 fimbriae with endogenous MR variant of the FimH protein increase systemic dissemination of *S*. Gallinarum and colonization of internal organs in comparison to the *S*. Gallinarum *fimH* knockout strain or the mutant expressing MS FimH variant from *S*. Enteritidis (Kuzminska-Bajor et al., 2012). Counter to typhoidal serovars, such as *S*. Gallinarum (Chappell et al., 2009), infections caused by non-typhoid *Salmonella* are associated with massive inflammation of the lamina propria and lymph nodes in the intestinal tract (Santos et al., 2009). Such intestinal inflammation can be exploited by* S.* Typhimurium to outcompete the intestinal microbiota, and promotes pathogen transmission (Thiennimitr et al., 2011). Interestingly, MS type 1 fimbriae not only promote adherence to gut mucosa, but also induce a strong immune response (Tchesnokova et al., 2011). Based on these findings, we speculated that MS type 1 fimbriae are advantageous in self-limiting gastrointestinal infections associated with a strong inflammatory host-response. In contrast, MR type 1 fimbriae, which lost mannose-specific adhesion, decrease *Salmonella* intestinal colonization and do not affect an inflammatory response (Kuzminska-Bajor et al., 2012). To further support our hypothesis, we investigated the involvement of MS type 1 fimbriae expressed by broad host-range *S.* Enteritidis in colonization of mice. Moreover, we evaluated the role of MS type 1 fimbriae in the induction of cytokine expression and secretion, the hallmark of an inflammatory response in intestinal cells. Based on our results, we propose that type 1 fimbriae may play an important role in the pathogenicity of *S*. Enteritidis and contribute to an intestinal inflammatory response.

## Materials and Methods

### Bacterial Strains and Growth Conditions

The *S.* Enteritidis strain (isolate no. 327) was collected from broiler chickens and described elsewhere (Kisiela et al., 2006). For optimal expression of type 1 fimbriae, bacteria were passaged five times in static LB broth at 37^∘^C. The presence of type 1 fimbriae was analyzed by flow cytometry using anti-FimH antibody (Kisiela et al., 2006) and hemagglutination assay.

### Mutant Construction

The construction of the *S.* Enteritidis mutant strain with the disruption of the *fimH* gene was performed according to Kuzminska-Bajor et al. (2012). Briefly, the wild-type *S.* Enteritidis strain was transfected with a DNA cassette containing the kanamycin resistance gene* kan^R^* amplified by PCR using plasmid pKD4 as template (kindly provided by The Coli Genetic Stock Center, Yale University, USA), and a pair of primers: 5′-cm_kan and 3′-cm_kan described previously (Kuzminska-Bajor et al., 2012). Primer 5′-cm_kan contained the additional sequence homologous to the 39 initial nucleotides of *fimH* gene, while the primer 3′-cm_kan contained the additional sequence corresponding to the terminal 39 nucleotides of this gene. For disruption of the *fimH* gene, bacteria transformed previously with pKD46 plasmid coding recombinase genes (kindly provided by The Coli Genetic Stock Center, Yale University, USA) were grown in LB broth supplemented with 100 μg/ml ampicillin and 10 mM arabinose. After homologous recombination with a linear DNA cassette, bacteria were plated on MacConkey agar plates containing kanamycin at a concentration of 50 μg/ml. The resulting *S.* Enteritidis mutant strain carrying *kan^R^* gene was named *S*. Enteritidis fimH::kan.

### Cells and Cell Culture

The mouse small intestine cell line (ICE-1) was a kind gift from InScreenex (InSCREENeX, Braunschweig, Germany; Schwerk et al., 2013). Cells were cultured in a defined IEC medium supplied by the manufacturer (InSCREENeX, Braunschweig, Germany).

### Invasion and Adhesion Assay

For invasion assay, *Salmonella*, after the fifth passage, were washed in PBS by centrifugation, resuspended in cell culture medium and adjusted by dilution to provide a MOI of 10:1 bacteria to host cells in culture wells of a six-well plate (∼8 × 10^5^ host cells and 8 × 10^6^ bacteria). Confluent monolayers were infected for 1 h, followed by an additional hour of incubation with culture media containing 50 μg/ml gentamicin (Sigma–Aldrich, St. Louis, MO, USA). After incubation, cells were washed three times with PBS and lysed with 0.1% Triton X-100 (Sigma–Aldrich) for 10 min. Bacterial suspensions were serially diluted with PBS and plated on LB-agar plates, incubated overnight at 37^∘^C, and the colonies were counted to calculate the CFU.

For adhesion assay, bacteria and ICE-1 cells were grown as described for the invasion assay. Infection was performed at a MOI of 100:1 *Salmonella* to host cells (∼8 × 10^5^ host cells and 8 × 10^7^ bacteria). After 2 h infection, cells were washed three times with PBS and lysed with 0.1% Triton X-100 (Sigma) for 10 min and plated as described for invasion assay. The percentage of dead cells was measured using bromophenol blue.

The adherence assay and invasion assays were also performed in the presence of 0.2 M D-mannose, by preincubation of bacteria with 0.2 M D-mannose for 1 h before exposition to the murine cells. Each assay was conducted in triplicate and was independently repeated at least three times.

### *In vivo* Experiments

The infectious potentials of wild-type *S.* Enteritidis and its *S*. Enteritidis fimH::kan mutant in mice were studied using bioluminescence imaging (BLI; Contag et al., 1995; Hutchens and Luker, 2007). BLI is a useful tool to understand differences in virulence potential of two different bacterial strains without the need to use death as an end point, thus reducing the suffering and number of animals used in comparison with conventional blind LD_50_ studies.

For this purpose, luciferase-expressing strains were obtained by the method of Riedel et al. (2007) Wild-type *S.* Enteritidis and mutated *S*. Enteritidis fimH::kan strain were electroporated with chromosomal integration vector p16Slux containing *lux* operon from *Photorhabdus luminescens* (kindly provided by Dr. Cormac Gahan, University College Cork, Ireland), and consisted of five genes *CDABE*. Three of them, *luxCDE*, encode a fatty acid reductase complex involved in synthesis of long-chain fatty aldehyde, which is an endogenous substrate for the luminescence reaction catalyzed by a heterodimeric luciferase, encoded by *luxAB*. Transformants were analyzed for bioluminescence using NightOwl 983 imaging system (Berthold Technologies, Bad Wildbad, Germany) and verified for the integration of p16Slux vector using PCR and primers 16Sint and 16SXHoI according to Riedel et al. (2007).

BALB/c female mice (Mossakowski Medical Research Centre, Polish Academy of Sciences, Warsaw, Poland), 6–8 weeks old, were infected by oral administration of bacteria suspended in 0.02 ml of PBS. The animal experiments were performed according to the International Animal Care Convention and were approved by the Local Ethic Committee for Animal Experimentation (Wrocław, Poland). After oral inoculation of mice with different doses (10^4^, 10^5^, and 10^7^ CFU/mouse) of wild-type *S.* Enteritidis and *S.* Enteritidis fimH::kan mutant, the animals (7/group) were monitored every 24 h for bioluminescent signals using NightOwl 983 imaging system. The control group was represented by mice inoculated with 0.02 ml of PBS. For whole-animal imaging, the mice were anesthetized with 5% isoflurane and anesthesia was maintained during imaging. Bioluminescence was recorded as pseudocolor images indicating light intensity (red being the most intense and blue the least intense). For co-localization of bioluminescence signals in animal bodies, pseudo-color images and gray-scale images of whole mice recorded with dimmed light were merged using Berthold WinLight 32 software.

Mice were sacrificed immediately after bioluminescent signals were detected, regardless of signal intensity. Our earlier studies indicated that the appearance of detectable bacterial luminescence was always followed by clinical manifestations of systemic disease, such as ruﬄed fur, lethargy, hunched posture, ataxia, tremor, eye discharge, and death within 24 h. In general, the symptoms of overt disease developed more quickly in mice with high signal intensities. After 21 days, mice with no detectable luminescent signals and no sign of infection were also sacrificed.

After the final image acquisition, the animals were euthanized and their spleen and livers dissected for determination of bacterial loads. For this purpose, the organs were homogenized in PBS, appropriately diluted and plated on MacConkey agar plates.

### Exposure of ICE-1 Cells to *Salmonella* Strains and FimH Protein

Confluent monolayers of ICE-1 cells were stimulated over a period of 1 or 4 h by co-incubation of *S.* Enteritidis wild-type or *S.* Enteritidis fimH::kan mutant at a MOI of 100:1 bacteria to murine cells or with recombinant *S.* Enteritidis FimH protein (Grzymajlo et al., 2010) at a concentration of 40 μg/ml in a IEC medium or with IEC medium alone. Afterward, cells were washed three times with PBS, trypsinized, and total RNA was isolated using Total RNA Mini Plus (A&A Biotechnology, Gdańsk, Poland) according to the manufacturer’s instructions.

In the case of secreted interleukin analysis, confluent monolayers of ICE-1 cells were stimulated over a period of 6 h by co-incubation with *S*. Enteritidis or *S*. Enteritidis fimH::kan mutant at a MOI of 100:1 bacteria to murine cells, or with a PBS as control, in IEC medium without supplementation. After 6 h, media were collected, centrifuged to remove bacteria and frozen at -20^∘^C. Secreted interleukins were determined by ELISA using Quantikine kits (R&D Systems, Minneapolis, MN, USA) or ELISA Ready-SET-Go (eBioscience, Inc., San Diego, CA, USA).

### Quantitative Real-Time PCR (qPCR)

First-strand cDNA was synthesized using the TranScriba kit (A&A Biotechnology, Gdańsk, Poland). The relative amounts of cytokine mRNAs were quantified by quantitative real-time PCR (qPCR) using the CFX thermocycler (BIO-RAD, Hercules, CA, USA) and the SYBR-green (Life Technologies, Carlsbad, CA, USA). *Actb* was used as a reference gene. Cytokine PCR primers are listed in **Table 1**. The reactions (total volume 20 μl) were performed under the following conditions: initial denaturation at 95^∘^C for 3 min, followed by 40 cycles of denaturation for 15 s at 95^∘^C, annealing for 25 s at 56^∘^C and elongation for 30 s at 72^∘^C. The specificity of the PCR was determined by melt-curve analysis for each reaction. The comparative Ct method was used for relative quantification of gene expression. All experiments were performed at least four times, and triplicate samples were analyzed in each experiment to confirm accuracy and reproducibility of qPCR. Fold change was measured over unstimulated cells set at 1.

**Table 1:** Primers used for quantitative real-time PCR (qPCR).

Tnfa-for	ACCCCTTTACTCTGACCCCT
Tnfa-rew	GGACTCTGAGCCATAATCCCC
Ifng-for	CCTGCGGCCTAGCTCTGAG
Ifng-rew	GCCATGAGGAAGAGCTGCA
Il-1b-for	ACAGGCTCCGAGATGAACAAC
Il-1b-rew	CCATTGAGGTGGAGAGCTTTC
Il-6for	GCCTTCTTGGGACTGATGCT
Il-6-rew	GACAGGTCTGTTGGGAGTGG
Il-10-for	CACAAAGCAGCCTTGCAGAA
Il-10-rew	GCTGATCCTCATGCCAGTCA
Il-12b-for	GGCTCTGGAAAGACCCTGAC
Il12b-rew	TGGAGCAGCAGATGTGAGTG
Actb-for	ACCACACCTTCTTACAATGAGC
Actb-rew	GATAGCACAGCCTGGATAGC

### Statistical Analysis

To confirm that the presence of MS type 1 fimbriae is a factor decreasing the colonization potential of *S.* Enteritidis, we compared the infection/bioluminescence-free-times of mice inoculated with wild-type *S.* Enteritidis or *S.* Enteritidis fimH::kan mutant strain. Infection-free curves were obtained using the method of Kaplan and Meier, and the significance of differences was determined by the Gehan–Breslow–Wilcoxon test.

Quantitative real-time PCR data, ELISA data, and colonization of internal organs were analyzed using the Mann–Whitney *U* test with non-gaussian distribution. A *P*-value of <0.05 was considered to be significant. All data were analyzed using the two-tailed tests.

## Results

### Characterization of *S*. Enteritidis *fimH* Gene Mutant

**FIGURE 1:**
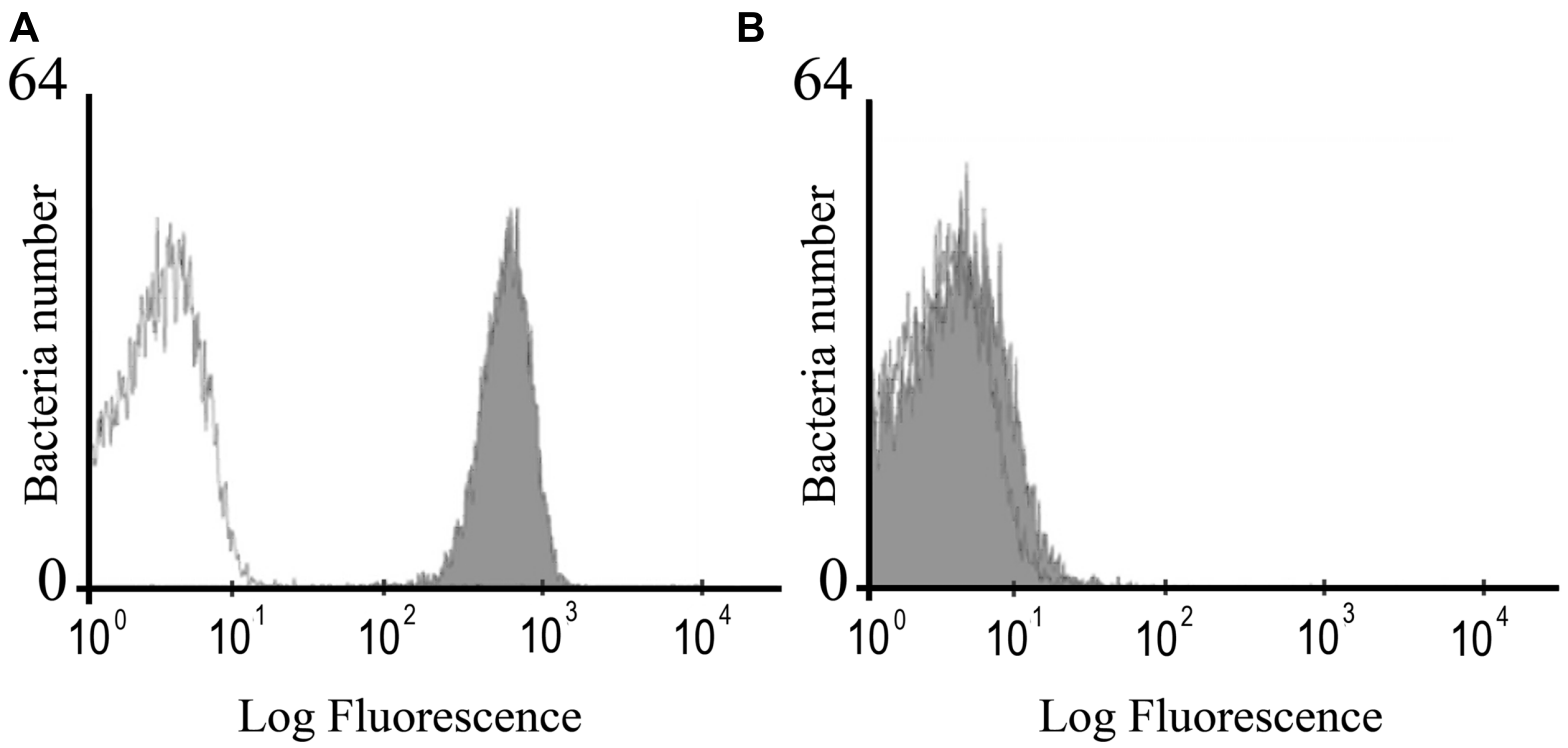
**Flow cytometry analysis of the FimH expression in wild-type *S*. Enteritidis **(A)** and *S*. Enteritidis fimH::kan mutant **(B)**.** The bacteria were stained with the polyclonal anti-FimH antibodies followed by secondary goat anti-rabbit FITC-conjugated antibodies (Sigma; filled gray peaks) or the secondary antibody only (open lines).

The mutant strain of* S.* Enteritidis with insertional inactivation of *fimH* gene had the same morphology and *in vitro* growth rate as the parental, wild-type bacteria. Phenotypic analysis of mutated bacteria revealed that they do not carry FimH protein on their surface in contrast to wild-type *S.* Enteritidis as shown by flow cytometry using specific anti-FimH antibody (**Figure 1** ) and hemagglutination assay using chicken erythrocytes and yeast cells.

**FIGURE 2:**
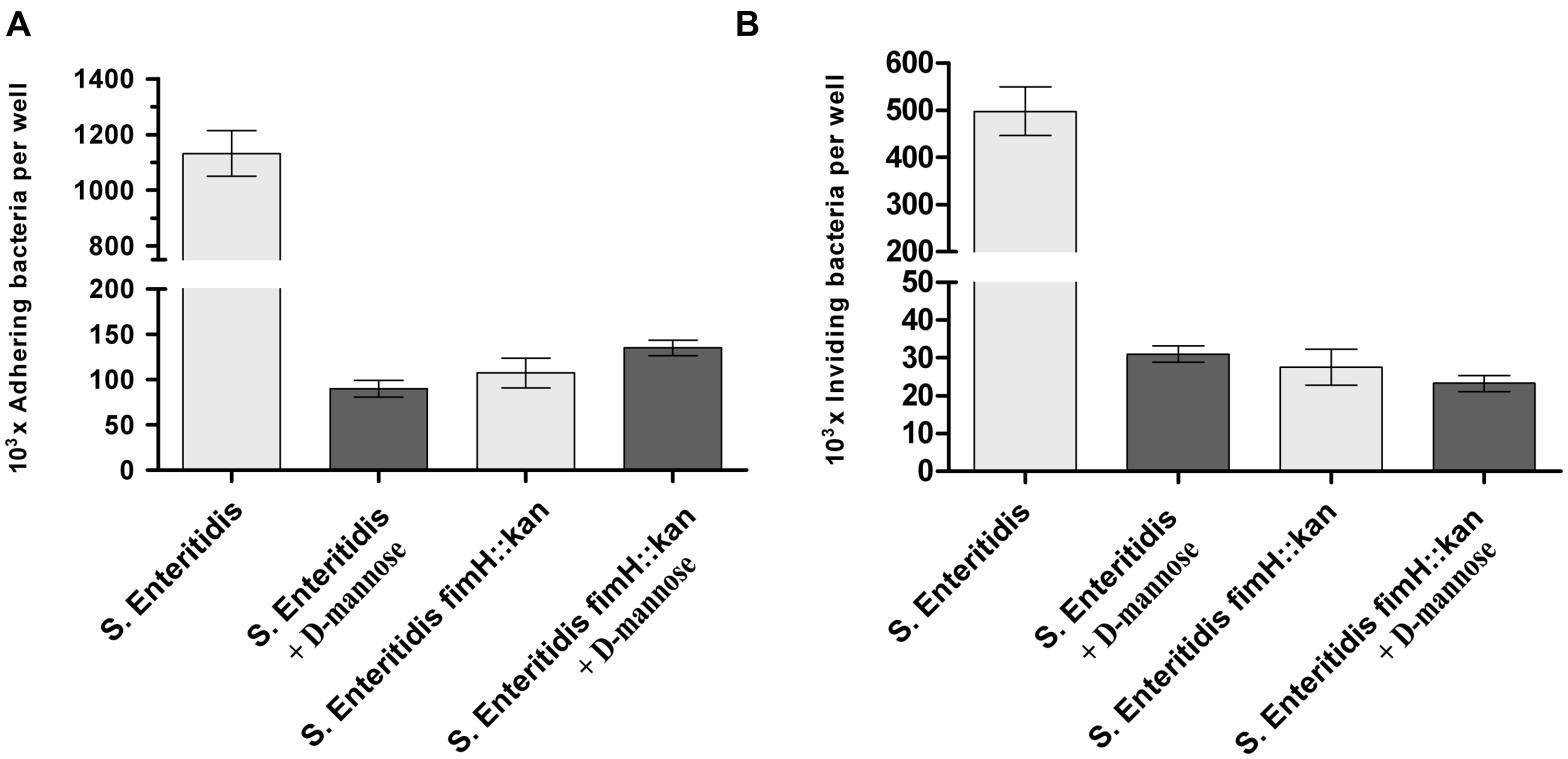
**Adherence **(A)** and invasion **(B)** of wild-type *S*. Enteritidis and *S*. Enteritidis fimH::kan mutant strain to murine intestinal ICE-1 cells, in the absence (light gray bars) and presence of 0.2 M D-mannose (dark gray bars).** Bacteria were grown statically at 37^∘^C in LB broth. For adhesion assay, bacteria (MOI 100:1), after five passages, were incubated for 1 h with cell monolayers growing in six-well plates. For invasion assay, cell monolayers growing in six-well plates were infected for 1 h with bacteria (MOI 10:1) after five passages, followed by an additional hour of incubation with culture media containing 50 μg/ml gentamicin. Data are means ± SD grown in triplicate assays, which were independently repeated at least three times.

When the functional activity of *S*. Enteritidis fimH::kan mutant was verified by the adherence of the bacteria to murine ICE-1 cells, the wild-type *S*. Enteritidis bound in significantly higher numbers (more than 10 times, *P* < 0.01) to eukaryotic cells than modified bacteria, and these interactions were inhibited by 0.2 M D-mannose (**Figure 2A**). Lower adhesion of *S.* Enteritidis fimH::kan mutant correlated well with its low invasion rate, which was more than 10 times lower than that for parental bacteria (*P* < 0.01; **Figure 2B**). Invasion efficiency calculated as invasion to adhesion ratio (invasion/adhesion) was about 26% for the mutant strain and about 46% for the wild-type strain. ICE-1 cells viability after 2 h exposition to *Salmonella* strains was above 96%.

### Colonization of Mice by Wild-Type *S.* Enteritidis and its *S.* Enteritidis fimH::kan Mutant Strain (Invasiveness *In vivo*)

**FIGURE 3:**
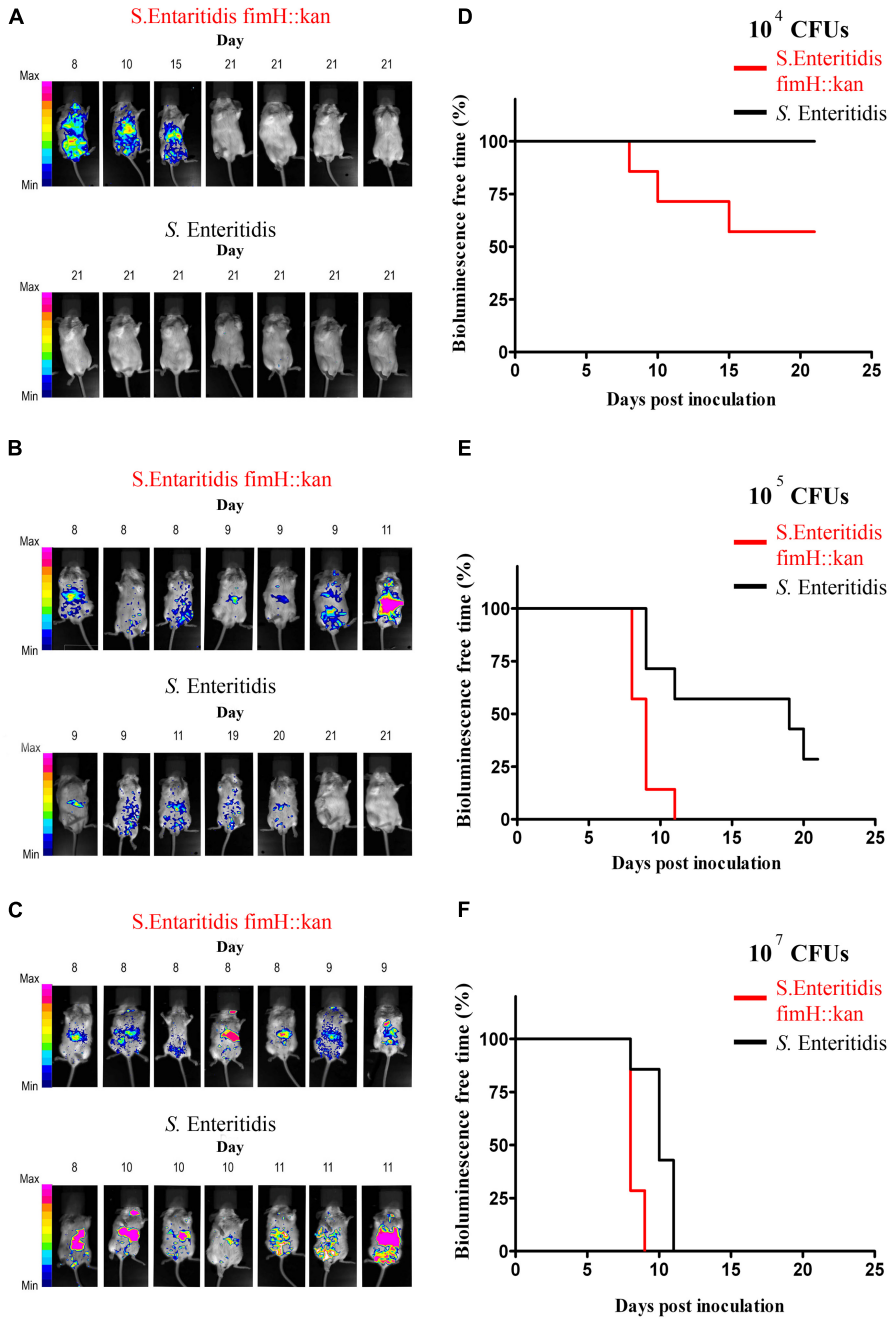
**Monitoring of wild-type *S*. Enteritidis and *S*. Enteritidis fimH::kan mutant infections in mice by whole-body bioluminescence imaging (BLI) and validation of type 1 fimbriae as a virulence factor of *S.* Enteritidis in mice.** BALB/c mice (7 per group) were inoculated *per os* with 10^4^* Salmonella* CFUs **(A)**, 10^5^* Salmonella* CFUs **(B)** and 10^7^ *Salmonella* CFUs **(C)** The presence of bioluminescence signals was monitored in animals every morning for 21 days after inoculation and intensity of bioluminescence emission is represented as a pseudocolor image. The animals were sacrificed immediately when the bioluminescent signal was observed. Infection-free-times, defined as bioluminescence-free-times were obtained using the method of Kaplan and Meier, and significance of differences was determined by the Gehan–Breslow–Wilcoxon test. Mice infected with 10^4^ **(D)**, 10^5^ **(E)**, and 10^7^ **(F)** *S*. Enteritidis fimH::kan mutant had significantly (*P* < 0.05) shorter infection-free times in comparison to animals inoculated with wild-type *S.* Enteritidis.

The results of *in vivo* studies are shown in **Figure 3**. Using BLI technology, striking differences in colonization patterns between wild-type *S.* Enteritidis expressing type 1 fimbriae and *S.* Enteritidis fimH::kan mutant with the deletion of the *fimH* gene were shown. Oral administration of 10^4^ *S.* Enteritidis fimH::kan CFUs caused infection in 43% (3 of 7) of the mice by day 15, however, after administration with wild-type bacteria all animals were free of *S.* Enteritidis on day 21 of the experiment (**Figure 3A**). In the case of mice infected with 10^5^ or 10^7^ doses of *S.* Enteritidis fimH::kan bacteria, bioluminescent signals were detected much earlier in mice infected with mutant strains of *S.* Enteritidis with no type 1 fimbriae, in comparison to wild-type bacteria (**Figure 3B,C**). The presence of *Salmonella*, detected by BLI, was confirmed by plating the spleen and liver homogenates on MacConkey agar plates. Bioluminescent signals were always accompanied by the presence of *S.* Enteritidis CFUs in the analyzed tissue samples. Nonetheless, luminescence intensity did not correlate with bacterial loads. The numbers of bacteria found in spleens and livers of mice, which develop bioluminescence signals, were comparable (about 9–11 Log_10_ CFU) regardless of the *Salmonella* strain (*S*. Enteritidis fimH::kan mutant *versus* *S*. Enteritidis wild-type) or the initial dose of bacteria. CFUs were not observed in livers and spleens isolated from mice which did not develop bioluminescent signals.

Mice infected with different doses of *S*. Enteritidis fimH::kan 10^4^, 10^5^, and 10^7^ CFUs had significantly (*P* < 0.05) shorter infection-free times in comparison to animals inoculated with wild-type *S.* Enteritidis (**Figure 3D–F**).

### Expression and Secretion of Cytokines in Murine ICE-1 Enterocytes in Response to *S*. Enteritidis Invasion is Dependent on the Presence of FimH Adhesin

**FIGURE 4:**
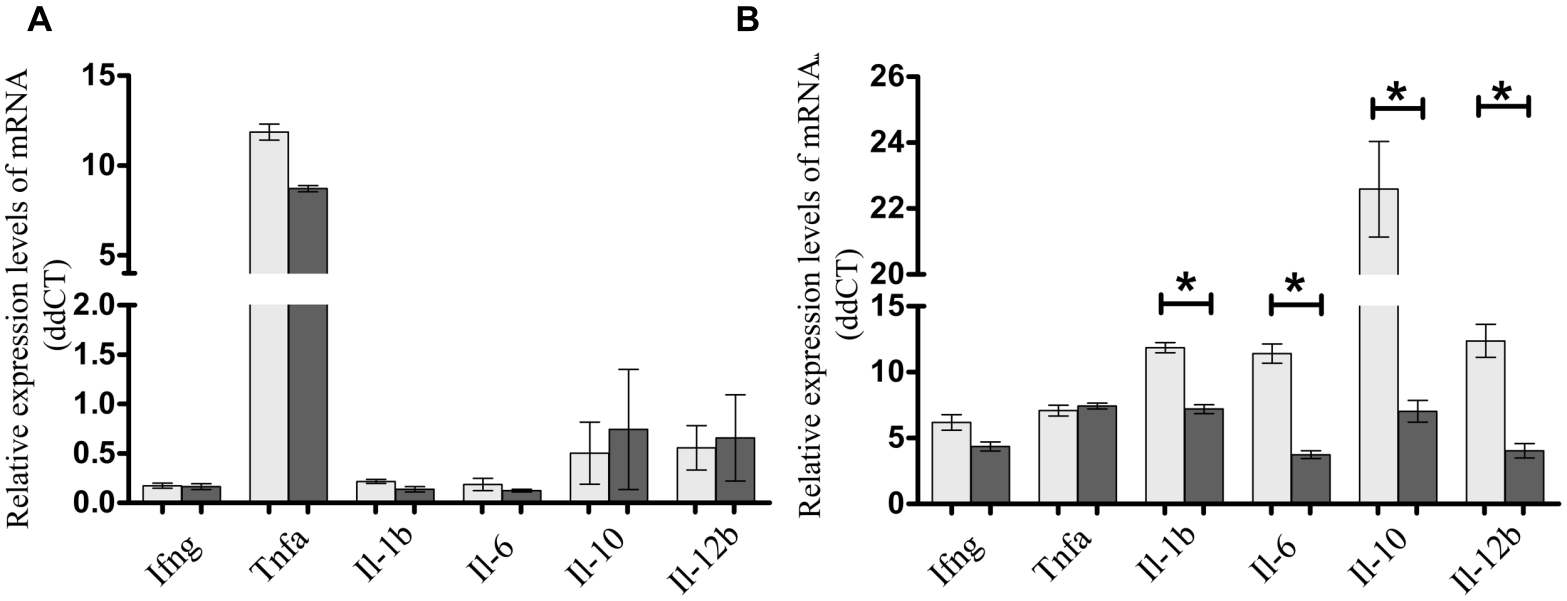
**Expression of *Ifng, Tnfa, Il-1b, Il-6, Il-10*, and *Il-12b* mRNAs in murine intestinal ICE-1 cells following 1 h **(A)** and 4 h **(B)** infection with MOI 1:100 of wild-type *S*. Enteritidis (light gray bars) and *S*. Enteritidis fimH::kan mutant strain (dark gray bars).** Real-time RT-PCR was used to analyze expression of cytokine mRNAs. Cytokine levels were normalized against *Actb*. Non-infected ICE-1 cells were assigned as a calibrator sample, and fold change was measured over unstimulated cells set at 1. The comparative Ct method was used for relative quantification of gene expression. Data represent the mean ± SD of four independent experiments. Triplicate samples were analyzed in each experiment to confirm accuracy and reproducibility of quantitative real-time PCR (qPCR). Values marked with ^∗^ differ significantly (*P* < 0.05).

**FIGURE 5:**
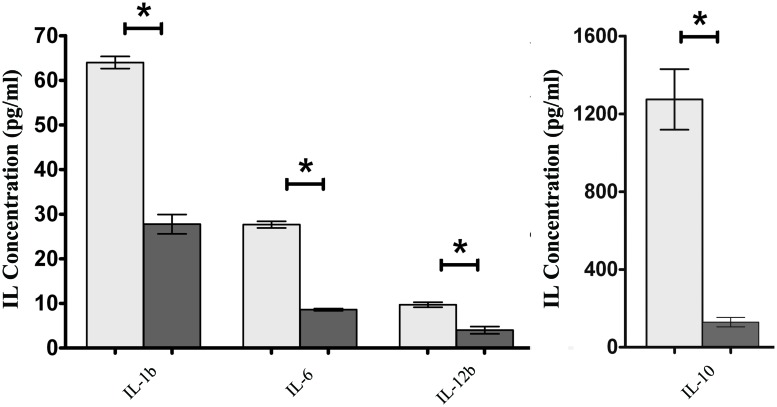
**Secretion of IL-1b, IL-6, IL-10, and IL-12b by ICE-1 cells exposed to wild-type *S*. Enteritidis (light gray bars) and *S*. Enteritidis fimH::kan mutant strain (dark gray bars) at MOI 100:1.** After 6 h incubation, the culture supernatants were collected and analyzed for the presence of interleukins by ELISA. Interleukin levels were normalized against unstimulated cells. Data represent the mean ± SD of four independent experiments, and were analyzed in each experiment to confirm accuracy and reproducibility of ELISA. Values marked with ^∗^ differ significantly (*P* < 0.05).

To assess if the presence of FimH adhesin and therefore type 1 fimbriae can affect the intestinal cell inflammatory response, *S.* Enteritidis fimH::kan mutant strain and wild-type *S*. Enteritidis were incubated with mouse intestinal epithelial ICE-1 cells for 1 or 4 h and expression of mRNA for cytokines: *Tnfa, Ifng, Il-1b, Il-6, Il-10*, and* Il-12b* was analyzed by qPCR. It was found that after 1 h of incubation, only the *Tnfa* level was significantly up-regulated in response to infection with each strain of *Salmonella* in comparison to untreated control cells (**Figure 4A**). After 4 h of incubation, the level of *Tnfa* mRNA decreased considerably, but at the same time the relative levels of other analyzed cytokines, with the exception of *Ifng*, increased highly in response to wild-type *S*. Enteritidis as well as the mutant strain (**Figure 4B**). Interestingly, in the case of pro-inflammatory cytokines *Il-1b, Il-6*, and* Il-12b* as well as anti-inflammatory *Il-10*, their mRNA levels were significantly higher (1.65x for *Il-1b*, *P* < 0.05; 2.75x for *Il-6*, *P* < 0.05; 3.06x for *Il-12b*, *P* < 0.05; 3.21x for *Il-10*, *P* < 0.05) after infection of murine cells by parental *S*. Enteritidis in comparison to non-fimbriated* S*. Enteritidis fimH::kan mutant strain. The results obtained on the level of mRNA by qPCR were further confirmed on the protein level. To do so, ICE-1 cells were incubated with *S*. Enteritidis fimH::kan mutant strain and wild-type *S*. Enteritidis for 6 h, the culture supernatants were collected and analyzed for the presence of IL-1b, IL-6, IL-10, and IL-12b by ELISA. Interleukin levels were normalized against unstimulated cells. In agreement with data obtained by qPCR, the secretion of IL-1b, IL-6, IL-10, and IL-12b by mouse intestinal epithelial ICE-1 was significantly higher (2.28x for IL-1b, *P* < 0.05; 3.09x for IL-6, *P* < 0.05; 10.07x for IL-10, *P* < 0.05; and 2,56x for IL-12b, *P* < 0.05) for the cell monolayers exposed to wild-type *S*. Enteritidis compared to *S*. Enteritidis fimH::kan mutant strain (**Figure 5**).

To assess, if soluble FimH protein has an impact on cytokine expression, mouse ICE-1 cells were incubated with purified and endotoxin free FimH protein from *S*. Enteritidis for 1 and 4 h. In both cases there were no significant changes in cytokine RNA expression levels compared to untreated control cells (data not shown).

## Discussion

In *Salmonella* infections the outcome of infection depends on the serovar causing disease. Non-typhoidal *Salmonella*, in immunocompetent individuals, are a common cause of self-limiting gastroenteritis and enterocolitis. Such serovars, e.g., *S*. Enteritidis and *S*. Typhimurium, usually infect many different animal species and therefore are called broad host-range. In contrast, infections caused by host-restricted* Salmonella* (e.g., *S*. Gallinarum, *S*. Typhi) lead to severe systemic disease, which is described as typhoid fever. It has recently been found that observed differences in clinical features correlate well with amino acid mutations in the FimH adhesins of type 1 fimbriae (Kisiela et al., 2012). Furthermore, the host-restricted (systemically invasive) *Salmonella* serovars express FimH adhesins with specific mutations which highly increase binding to mannose ligands (*S*. Typhi, *S*. Paratyphi C, *S*. Dublin, and some isolates of *S*. Choleraesuis) or cause complete loss of mannose-binding activity (*S*. Gallinarum, *S*. Paratyphi B, *S*. Choleraesuis). On the other hand, broad host-range (systemically non-invasive) *Salmonella* express MS allelic variants of FimH adhesins with low-adhesive properties (Kisiela et al., 2012). The results of these genetic studies are consistent with our previous studies on the pathogenicity of *S*. Gallinarum expressing MS FimH adhesin from *S*. Enteritidis (Kuzminska-Bajor et al., 2012). In chick model, *S*. Gallinarum expressing MS FimH adhesin from *S*. Enteritidis colonized cecal tonsils and bursa of Fabricius less effectively and invaded the spleen and liver in smaller numbers than wild-type *S*. Gallinarum, indicating the involvement of these adhesive structures in the outcome of infection. Elaborating from our previous studies, we proposed that MS variants of FimH are advantageous in gastrointestinal infections, in contrast to MR FimH variants which decrease intestinal colonization and promote their systemic spreading. To further support our hypothesis, we carried out *in vivo* studies using mice infected with wild-type *S.* Enteritidis and its mutant strain unable to produce FimH adhesin using BLI technology, which allows real-time monitoring of bacterial infections. Despite the fact that bioluminescence signals were always accompanied by the presence of *S*. Enteritidis CFUs, in our experimental conditions luminescence intensity poorly correlated with bacterial loads. The intensity of bioluminescence signals depends strongly on the localization of bacteria and variable positioning of the intestine within the abdomen, resulting in inconsistent signal intensities. Furthermore, strong attenuation of light production and bioluminescence emission is due to pigmentation of fur and organs such as liver and spleen. Due to such limitations, BLI is usually defined as semi-quantitative approach to study internal organs colonization. Nonetheless, it is an excellent technique to predict and confirm systemic disease in single mouse.

We observed that the loss of MS FimH adhesin correlated well with the highly increased colonization of mice by these bacteria. The appearance of the FimH-devoid mutant strain was observed much earlier than wild-type *Salmonella*, and mice infected with 10^4^–10^7^ S. Enteritidis fimH::kan CFUs had significantly (*P* < 0.05) shorter infection-free time than animals inoculated with wild-type *S.* Enteritidis. These results give direct evidence that *S.* Enteritidis expressing MS type fimbriae is less invasive in mice and has less potential for systematic dissemination than bacteria devoid of these organella.

Following oral infection, intestinal epithelial cells are the first barrier to be crossed by *Salmonella* to invade the ileal and colonic mucosa and spread to other organs (liver, spleen). The innate immune system, which includes intestinal epithelial cells, is primarily responsible for the detection of invading *Salmonella* and then the induction of inflammation to overcome or at least to restrict bacterial infection. Interestingly, recent data strongly suggest that intestinal inflammation is a positive factor which helps the luminal fraction of* S.* Typhimurium to survive and expand, and can be exploited to outcompete the intestinal microbiota. It also promotes pathogen transmission (Stecher et al., 2007; Barman et al., 2008; Lawley et al., 2008; Santos et al., 2009). So far nothing is known, in contrast to other virulent factors such as type III secretion systems 1 and 2 (T3SS-1 and T3SS-2) or flagella (Tsolis et al., 1999; Schmitt et al., 2001), about the involvement of type 1 fimbriae in the induction of an intestinal inflammatory response by *Salmonella*. To approach this problem, we studied changes in expression of selected cytokines (*Tnfa, Ifng, Il-1b, Il-6, Il-10*, and* Il-12b*), known to be involved in *Salmonella* infections (Kincy-Cain et al., 1996; Pie et al., 1996; Gulig et al., 1997; Everest et al., 1998; Bao et al., 2000; Stadnyk, 2002; Collado-Romero et al., 2010; Awoniyi et al., 2012; de Jong et al., 2012) in mouse intestinal epithelial cells exposed to wild-type *S*. Enteritidis and *S.* Enteritidis fimH::kan mutant devoid of type 1 fimbriae, since it is now widely accepted that increased expression of cytokines is the hallmark of intestinal inflammation (Eckmann and Kagnoff, 2001; Mavris and Sansonetti, 2004).

In accordance with the above data, we found that infection of murine intestinal epithelial cells with *S*. Enteritidis resulted in an increase of gene expression for all analyzed cytokines. However, our study also revealed profound quantitative differences in the levels of cytokine mRNA expression when cells were infected with wild-type *S*. Enteritidis in comparison to the non-fimbriated *S*. Enteritidis fimH::kan mutant strain. Using qPCR, we demonstrated that the expression of *Il-1b*, *Il-6*, *Il-10*, and* Il-12b* was significantly higher in cells infected with wild-type *S*. Enteritidis compared to cells infected with the mutant strain. Moreover, we found that increased expression on the mRNA level, was accompanied by highly increased secretion of all four interleukins studied. Based on these results and data from *in vivo* studies, we concluded that MS type 1 fimbriae expressed by non-typhoidal *Salmonella* serovars play an important role in the induction of inflammatory processes during intestinal infections, helping fimbriated *Salmonella* to survive in such an environment, and limiting infection to the gastrointestinal tract. The question remains, what are the mechanisms by which MS type 1 fimbriae increase the expression of cytokine genes in intestinal epithelial cells. Based on the observation that type 1 fimbriae-mediated adhesion correlates strongly with the invasive potential of *S*. Enteritidis, we suggest that MS FimH adhesins from broad host-range *Salmonella* with shear-activated properties (Sokurenko et al., 2008), allow bacteria to roll along the intestinal wall and in this way enable them to invade in higher numbers the enterocytes and stimulate more effectively the expression of various cytokines involved in the induction of inflammation. These in turn promote the luminal outgrowth of the remaining bacteria, which adhere to intestinal epithelial cells using type 1 fimbriae. This hypothesis is supported by our finding that the binding of purified FimH adhesin did not change the expression of cytokine genes, which excludes the possibility of direct involvement of type 1 fimbriae in the activation of these genes.

In summary, from our present studies it appears to be clear that fimbriated wild-type *S.* Enteritidis is less virulent than the non-fimbriated *S.* Enteritidis mutant strain. Since the presence of MS-type 1 fimbriae significantly affects the expression and secretion levels of several cytokines in mouse enterocytes, we propose that type 1 fimbriae contribute to the induction of intestinal inflammation during *Salmonella* invasion. At the same time, the presence of MS type 1 fimbriae allows bacteria to adhere to inflammatory epithelium, which become the ideal environment for the growth of this pathogen. Therefore, we speculate that type 1 fimbriae are important factors limiting the dissemination and colonization by *S.* Enteritidis and other non-typhoid serovars to the intestinal tract.

## Conflict of Interest Statement

The authors declare that the research was conducted in the absence of any commercial or financial relationships that could be construed as a potential conflict of interest.
